# MRI-detected intraosseous bone marrow edema recedes after effective therapy of periodontitis

**DOI:** 10.1007/s00330-023-10327-6

**Published:** 2023-10-19

**Authors:** Julian Schwarting, Florian Andreas Probst, Magdalena Griesbauer, Teresa Robl, Egon Burian, Benedikt Wiestler, Teresa Brunner, Yoana Malenova, Caspar Bumm, Matthias Folwaczny, Monika Probst

**Affiliations:** 1 Department of Diagnostic and Interventional Neuroradiology, Klinikum rechts der Isar, Technical University, Ismaninger Str. 22, 81675 Munich, Germany; 2 Department of Oral and Maxillofacial Surgery and Facial Plastic Surgery, University Hospital, Ludwig-Maximilians-University, Munich, Germany; 3 Department of Diagnostic and Interventional Radiology, Klinikum rechts der Isar, Technical University, Munich, Germany; 4 Department of Restorative Dentistry and Periodontology, University Hospital, Ludwig-Maximilians-University, Munich, Germany

**Keywords:** Magnetic resonance imaging, Periodontal disease, Periodontitis

## Abstract

**Objectives:**

T2 STIR MRI sequences can detect preclinical changes associated with periodontal inflammation, i.e. intraosseous edema in the tooth-supporting bone. In this study, we assessed whether MRI can be used for monitoring periodontal disease.

**Material and methods:**

In a prospective cohort study, we examined 35 patients with periodontitis between 10/2018 and 04/2019 by using 3D isotropic T2-weighted short tau inversion recovery (STIR) and Fast Field Echo T1-weighted Black bone sequences. All patients received standardized clinical exams before and three months after non-surgical periodontal therapy. Bone marrow edema extent was quantified in the STIR sequence at 922 sites before and after treatment. Results were compared with standard clinical findings. Non-parametric statistical analysis was performed.

**Results:**

Non-surgical periodontal treatment caused significant improvement in mean probing depth (*p* < 0.001) and frequency of bleeding on probing (*p* < 0.001). The mean depth of osseous edema per site was reduced from a median [IQR] of 2 [1, 3] mm at baseline to 1 [0, 3] mm, (*p* < 0.001). Periodontal treatment reduced the frequency of sites with edema from 35 to 24% (*p* < 0.01).

**Conclusion:**

The decrease of periodontal bone marrow edema, as observed with T2 STIR MR imaging, is indicative of successful periodontal healing.

**Clinical relevance statement:**

T2 STIR hyperintense bone marrow edema in the periodontal bone decreases after treatment and can therefore be used to evaluate treatment success. Furthermore, MRI reveals new options to depict hidden aspects of periodontitis.

**Key Points:**

• *T2 STIR hyperintense periodontal intraosseous edema was prospectively investigated in 35 patients with periodontitis before and after treatment and compared to clinical outcomes.*

• *The frequency of affected sites was reduced from 35 to 24% (p < 0.001), and mean edema depth was reduced from a median [IQR] of 2 [1, 3] mm at baseline to 1 [0, 3] mm 3 months after treatment. (p < 0.001).*

• *T2 STIR sequences can be used to monitor the posttreatment course of periodontitis.*

**Supplementary Information:**

The online version contains supplementary material available at 10.1007/s00330-023-10327-6.

## Introduction

Periodontitis is an inflammatory disease that results in the resorption of the tooth-supporting alveolar bone and the destruction of the periodontal ligament. Periodontal attachment loss is diagnosed clinically based on bleeding on probing and the probing pocket depth [1]. Complementary to clinical findings, a panoramic radiograph is commonly used to determine and visualize the disease-associated bone loss in the diagnosis and monitoring of periodontitis. Computed tomography and cone beam computed tomography provide three-dimensional diagnostic views of the tissue defects associated with periodontitis; however, it is difficult to visualize early inflammatory changes by X-ray-based techniques in soft tissue compartments and the bone marrow [2].

A combination of a 3D T1 Black bone with a 3D T2 short tau inversion recovery (STIR) sequence was demonstrated to outline not only already apparent periodontitis-associated tissue defects but also inflammatory intraosseous changes [3]. The detected intraosseous edema correlated with the severity of the disease, i.e., the probing pocket depth and the presence of bleeding on probing, indicating active gingival and/or periodontal inflammation.

According to these data, MRI-based findings provide additional information in diagnosing periodontitis and gingival inflammation. Yet, it remained unclear if these intraosseous changes could be influenced by periodontal treatment measures. This question was to be investigated in the presented study in a prospective cohort of patients with periodontitis intraosseous inflammatory changes who were examined immediately before and 3 months after standardized non-surgical periodontal treatment with 3D T1 Black bone and 3D T2 STIR sequences.

## Methods

### Study design

This prospective cohort study was designed in accordance with STROBE guidelines [4]. The trial received an institutional review board approval (Technical University Munich: Ref.-No.185/18 S and Ludwig-Maximilians-University Munich: Ref.-No. 18-657) and was retrospectively registered at the DRKS (German Clinical Trials Register, DRKS00020761).

Seventy-six patients presenting at the Department of Periodontology, Ludwig-Maximilians-University Munich were screened from October 2018 to April 2019. 42 patients fulfilled the inclusion criteria:

1. Clinical evidence of periodontal disease, defined according to most recent guidelines: Interdental attachment loss or buccal/oral attachment loss of ≥ 3 mm with pocketing ≥ 3 mm not caused by non-periodontitis reasons at ≥ 2 non-adjacent teeth.
2. At least 12 teeth in situ with > 30% of teeth periodontally affected
3. No previous endodontic or surgical treatment in the investigated tooth 12 months before the first scan and no signs of occlusal stress.
4. Absence of current relevant medical treatments interfering with bone marrow edema, i.e., antiresorptive and/or antiangiogenic drugs.
5. No contraindication for MRI imaging
6. Written informed consent.

**Fig. 1:**
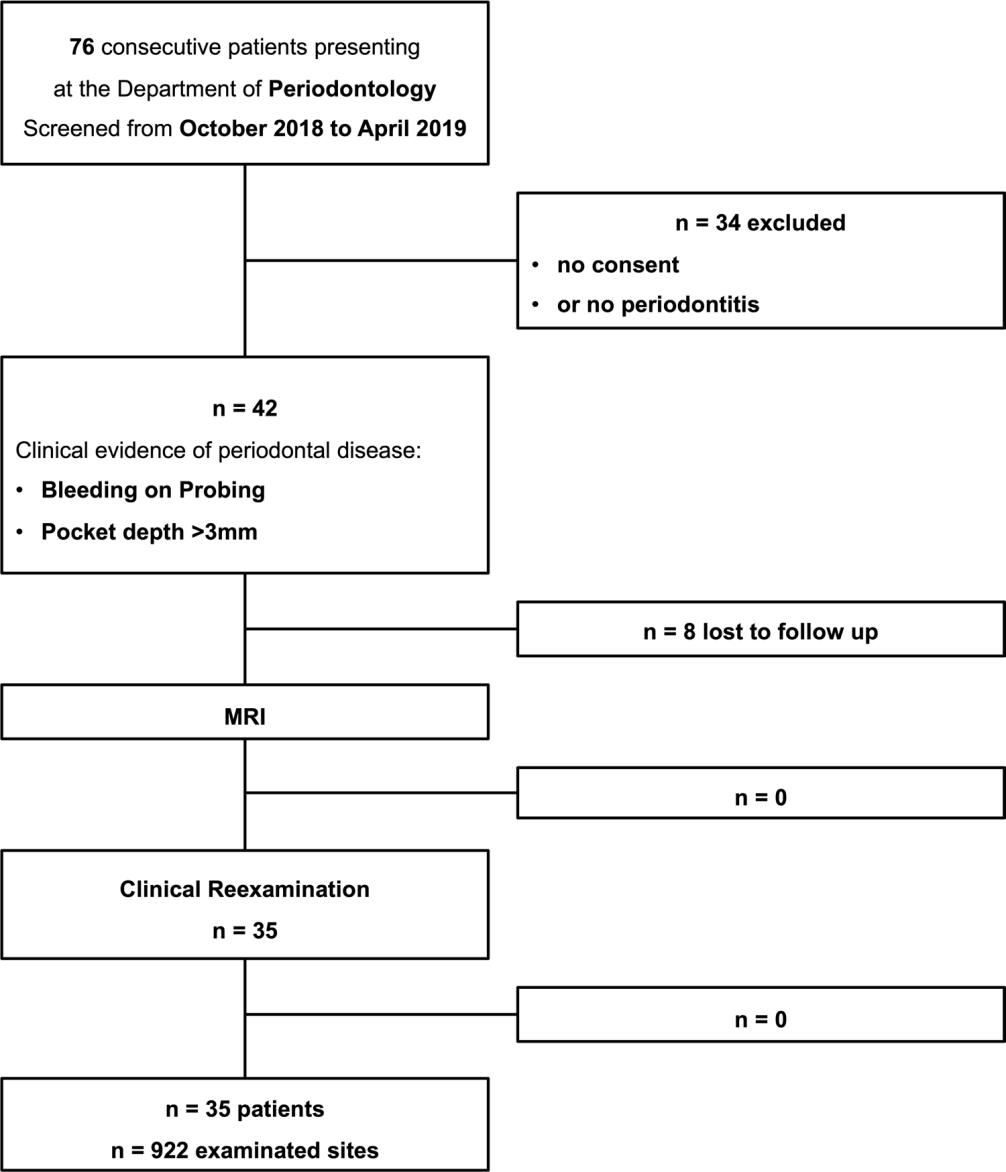
Flow diagram of study participants

After an initial standardized clinical staging and MRI, all patients received standardized non-surgical periodontal treatment, including oral hygiene instructions, elimination of plaque retentive factors, including removal of restorations with poor marginal adaption and supra- and subgingival instrumentation with hand instruments (Gracey curettes, HuFriedy) and sonic scalers (SonicFlex, KAVO Dental) supplemented by systemic antimicrobial treatment, if indicated [5]. Three months after completion of active periodontal therapy, patients were reevaluated with a complete periodontal examination and a follow-up MRI employing the same scanner and imaging protocol as used for the baseline MRI. Seven patients did not present for the second clinical examination and MRI (Fig. 1).

Clinical findings were not available to the MRI examiners, nor were the results of MR imaging available to clinical examiners.

### MR imaging

Patients were examined with a 3-T MRI scanner (Elition, Philips Healthcare) at the Department of Diagnostic and Interventional Neuroradiology, Technical University Munich, using a 16-channel Head Neck Cervical Spine Array. No additional surface coil was used. Patients were positioned head-first in a supine position. The sequencing protocol consisted of a short survey for sequence position planning (acquisition time 0:39 min), a 3D isotropic T2-weighted STIR sequence, and a 3D isotropic Fast Field Echo (FFE) T1-weighted Black bone sequence (acquisition time 5:31 min). Detailed imaging parameters are summarized in the Suppl. Tab. S1. 3D T1 bone sequence served to determine changes within the tooth-supporting alveolar bone associated with periodontitis.

### MR image analysis

3D T1 Black bone and 3D T2 STIR sequences of both time points were co-registered using the open-source software “elastix” [6, 7]. Co-registration was necessary to ensure that VOIs and linear measurements were located at identical positions in both sequences within the alveolar ridge in all quadrants.

**Fig. 2:**
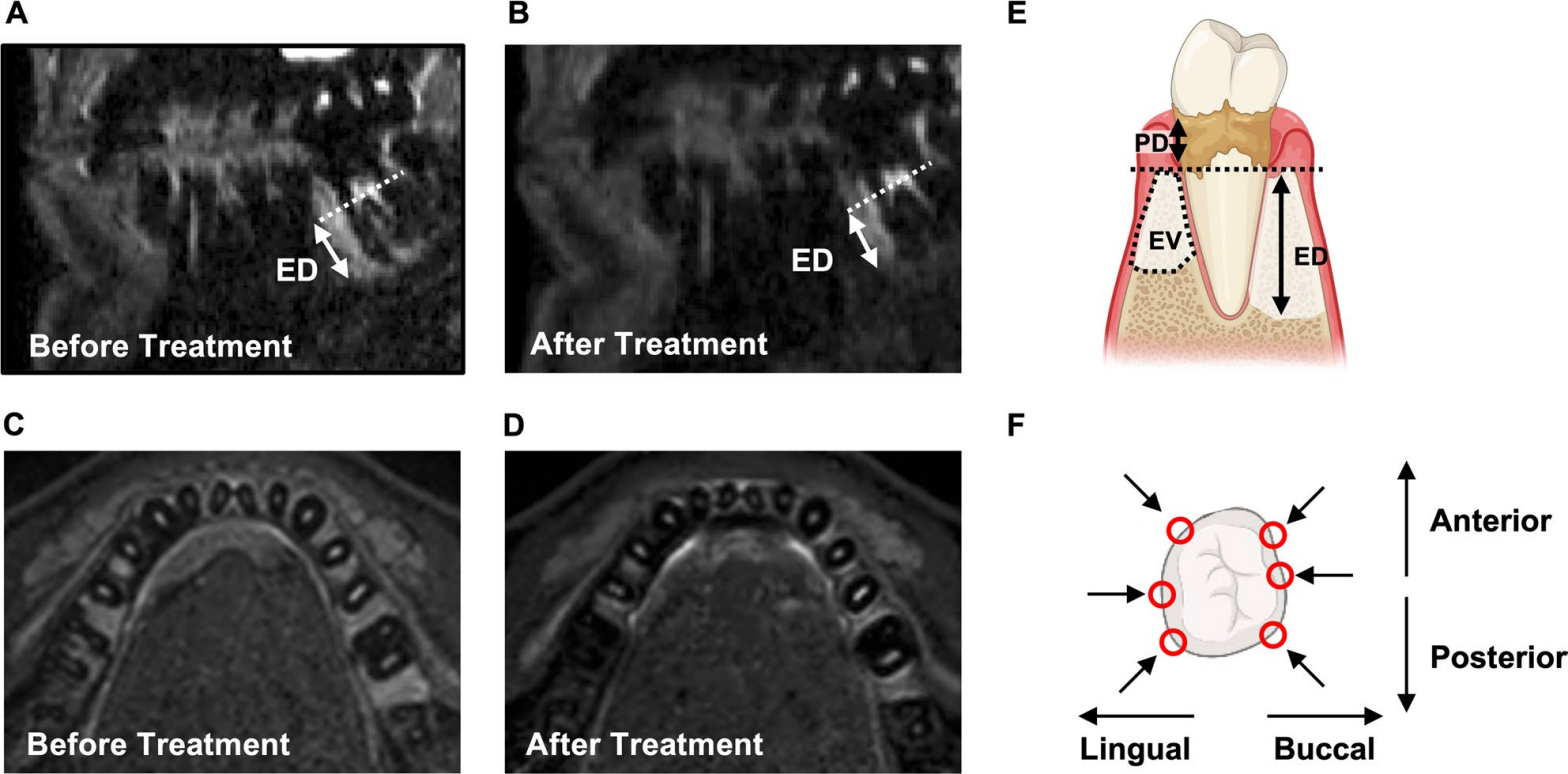
Sagittal (**A, B**) and axial (**C, D**) T2 STIR images of the same patient before and three months after non-surgical treatment of periodontal disease shows intraosseous edema. Quantification was performed in linear bone marrow edema depth (ED) measurements and volumetric measurements of bone marrow edema volume (EV). This was correlated with probing depth (PD, **E**), measured in 6 sites in Molars (**F**). Data is shown in Table 3

The linear extent of the bone marrow edema (edema depth, ED) was measured within the bone in apical-coronal direction before and after treatment in molar teeth in 6 specific sites: Lingually and buccally, each anterior, mid, and posterior (Fig. 2F).

Osseous edema was thereby primarily measured in 3D T2 STIR sequence. To ascertain that measurements were only within the bone, measurements were then transferred to the co-registered 3D T1 Black bone sequence. A maximum of 6 sites were evaluated for every tooth, simultaneously with the standardized clinical probing methodology. Maximum edema depth was used in every tooth for comparison and statistical testing.

For volumetric measurements, alveolar ridges were segmented separately for each quadrant based on anatomical information retrieved from 3D T1 Black bone sequences. In quadrants with initial edema, two subtraction images were calculated: 1. Pre-treatment minus post-treatment imaging, showing edema reduction, and 2. Post-treatment minus pre-treatment subtraction showing increasing edema. These subtraction cards contained the voxels for which the analysis was applicable. As the voxel size is defined with isotropic 0.65 mm, the edema volume could be calculated in mm^3^. Signal intensities of each voxel were compared to the mean signal intensity in the previously selected volume of bone marrow. Voxels showing a signal of > 2 standard deviations (SD) in comparison to the mean signal intensity of the selected bone marrow indicated edema.

**Fig. 3:**
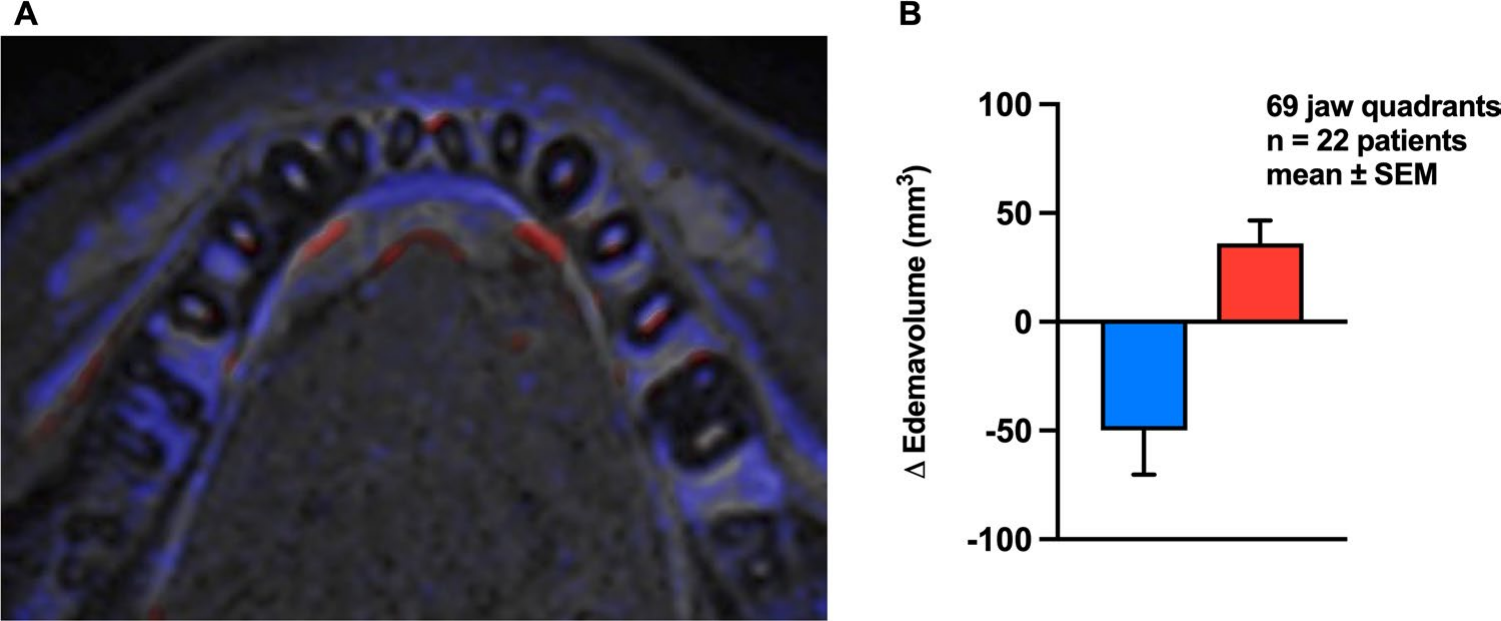
**A** Exemplary T2 STIR subtraction map of bone edema of a single patient before and after treatment. Bone marrow edema reduction (blue) and new edema occurrence (red) were visualized using the Longitudinal Brain Imaging algorithm (Philips IntelliSpace Portal). **B** Quantification of edema volume changes per jaw quadrant in 22 patients

To visualize the changes in edema volume in Fig. 3A, we used the software package “IntelliSpace Portal Longitudinal Brain Imaging” (Koninklijke Philips N.V.).

All image analysis was performed by a neuroradiologist (MD with 12 years of work experience) and by a dentist and radiologist (MD, DMD with 7 years of radiological work experience). Examiners were blinded for the clinical status of the patients. In case of severe artifacts due to metallic restorations or movement artifacts, single teeth were excluded from further analysis.

### Statistical analysis

Sample size calculation was performed using the G*Power Calculator (version 3.1), revealing a minimum sample size of 35 patients’ teeth to reach a power of 0.9 (effect size d =1, Alpha error probability 5%). We increased the number of included patients by 10% to account for potential dropouts. SPSS software version 26.0 (SPSS Inc) was used for all statistical tests.

The normal distribution of data was tested using the Kolmogorov-Smirnov procedure. If the acquired data was not normally distributed, we showed the Median [IQR]. Wilcoxon test was applied to compare dependent variables (i.e. before and after treatment). Mann-Whitney test was used for independent variables. The absolute numbers and relative frequencies within each experimental group are presented for categorical data. Comparisons between groups were performed with the Pearson chi-square test with Yates correction. If appropriate (comparison of two groups), test procedures were two-tailed. *p* values < 0.05 were considered significant. Patients lost in follow-up were excluded from the analysis.

## Results

### Patient cohort

The study cohort initially comprised 42 patients (mean age 58 ±14.6 years; age range 20–90 years, male to female ratio 25:17) diagnosed as localized in 8 (19%) patients and as generalized in 34 (81%) of cases. 35 patients returned for clinical and imaging (MRI) reevaluation representing 170 teeth in 922 observation sites. Detailed clinical information of the included patients is shown in Table 1.

**Table 1:** Patient characteristics

Patient characteristics	
Age	58 (15)
Age range	20–90
Female	*n* = 16
Generalized Periodontitis	*n* = 30
Smoker (>10 pack years)	*n* = 4
PA caused toothloss	*n* = 7
Familiar predisposition	*n* = 5
Diabetes mellitus	*n* = 2
Arterial hypertension	*n* = 5
Osteoporosis	*n* = 1
Tooth status of included patients	
Avg. teeth per patient	*n* = 24
Avg. molar teeth per patient	*n* = 6
Avg. affected molars per patient	*n* = 5
Excluded teeth per patient (movement artifacts)	2%
Excluded teeth per patient (susceptibility artifacts)	8%

### Clinical findings following non-surgical periodontal treatment

After non-surgical periodontal treatment, we observed a positive treatment response. Considering all observed sites, the mean probing pocket depth was reduced from a median of 3 [3, 5] to a median of 3 [3, 4] (*p* < 0.001). Treatment effects varied thereby between pocket depths: While we observed no significant difference in the median depth in pockets initially measured with ≤ 3 mm, significant differences could be detected in deeper pockets with initial depths of ≥ 4 mm (Table 2).

**Table 2:** Clinical parameters at single sites before and after periodontal treatment

Clinical Results	PPD before treatment	PPD after treatment	***p***
all sites (*n *= 922)	3 [3, 5] mm	3 [3, 4] mm	< 0.001
PPD ≤ 3 mm without BOP (*n* = 319)	3 [1, 3] mm	3 [1, 3] mm	0.39
PPD ≤ 3 mm with BOP (*n* = 166)	3 [3] mm	3 [3] mm	0.58
PPD 4-5 mm (*n* = 416)	4 [4, 5] mm	3 [3, 4] mm	< 0.001
PPD ≥ 6 mm (*n* = 123)	6 [6, 7] mm	5 [4, 6] mm	< 0.001
	Before treatment n (%)	After treatment n (%)	
PPD ≤ 3 mm	591 (52%)	631 (57%)	0.029*
PPD 4-5 mm	379 (33%)	347 (31%)	0.029*
PPD ≥ 6 mm	170 (15%)	132 (12%)	0.029*
with BOP	508 (45%)	312 (28%)	< 0.001

Data in parenthesis are IQR. PPD = Probing pocket depth. BOP = Bleeding on probing * Comparison between categories by chi-square test

Also, relative frequencies changed after treatment: Here, we were able to detect fewer pockets with a probing depth ≥ 4 mm and more shallow pockets (*p *< 0.001, Table 2).

The overall frequency of sites with gingiva bleeding on standardized probing, and bleeding on probing (BOP) decreased from 55 to 27% (*p* < 0.001), in line with these findings.

### Findings in MR images

MR images in parasagittal and axial orientation are shown in Fig. 2 A–D. Overall mean osseous edema depth (ED) measured as shown in Fig. 2E was reduced from an initial median of 2 [1, 3] mm at baseline to a median of 1 [0, 3] mm 3 months after treatment (*p* < 0.001, Fig. 2A–D, Table 3).

**Table 3:** Edema extent at single sites before and after periodontal treatment

MRI Results	Affected sites before treatment	Affected sites after treatment	*p*
All sites (*n* = 922)	321 (35%)	278 (24%)	< 0.001
	ED before treatment	ED after treatment	
All sites with initial edema (n = 321)	2 [1, 3] mm	1 [0, 2] mm	< 0.001
PPD ≤ 3 mm, without BOP (*n* = 31)	2 [1, 3] mm	2 [1, 3] mm	0.015
PPD ≤ 3 mm, with BOP (*n* = 33)	1 [1, 3] mm	1 [0, 3] mm	0.002
PPD 4-5 mm (*n* = 65)	1 [1, 2] mm	1 [0, 1.5] mm	< 0.001
PPD ≥ 6 mm (*n* = 102)	2 [2, 5] mm	1 [0, 3] mm	< 0.001

*Data in parenthesis are IQR. ED = edema depth. PPD = Probing pocket depth. BOP = Bleeding on probing*

Edema depth was significantly reduced in shallow (PPD ≤ 3 mm; no BOP: *p *= 0.015; BOP: *p* = 0.002), medium (PPD 4–5 mm; *p* < 0.001), and deep sites (PPD ≥ 6 mm; *p* < 0.001), independently of the occurrence of bleeding on probing (Table 3).

The overall proportion of sites with MR-detectable osseous edema was reduced in all sites from 35% before to 28% after treatment (*p* < 0.001, Table 3).

### Volume-based measurements

By comparing co-registered images of the same patient before and after treatment, reductions in edema volume as well as an occurrence of new edema could be observed in some locations (Fig. 3A). In the 69 affected jaw quadrants of 22 patients with initial edema we detected a mean reduction of 49 mm^3^ with a Standard error of the mean (SEM) of 20 mm^3^/ jaw quadrant as well as the new occurrence of 36 ± 10 mm^3^/ jaw quadrant after therapy by manual segmentation of subtracted images (Fig. 3B).

## Discussion

In the current study, we performed 3D T1 black bone and 3D STIR MR imaging in 35 patients with the clinical diagnosis of generalized periodontitis before and three months after standard non-surgical treatment. After therapy, we observed 11% fewer sites with periodontal edema (*p* < 0.01), and edema depth was reduced from initially 2 mm to 1 mm (*p* < 0.01). Changes were most prominent in sites with originally deep edema (≥ 6 mm) and were independent of gingivitis (bleeding on probing). Volumetric quantification showed changes in edema extend between both time points. Edema depth could therefore be the first MR radiographic parameter to monitor disease activity and bone affection in periodontitis.

Until now, periodontal disease activity is primarily monitored using clinical parameters, i.e., probing pocket depth, attachment level, and "bleeding on probing.” Dynamics in periodontal pocket depths have a limited diagnostic accuracy as periodontal inflammation can also heal regardless of remaining periodontal pockets [8–10].

Bleeding upon probing can indicate persistent and/or recurring periodontal disease activity and further attachment loss. However, gingivitis must not be associated with periodontal inflammation, which is associated with the actual progression of tissue destruction. Interestingly, we observed less edema in sites with periodontal pockets ≤ 3 mm with bleeding on probing in comparison to sites without. A potential explanation for this could be the healing of gingivitis while the osseous manifestations of periodontitis persist and progress. However, this hypothesis must be proven histologically in a follow-up study.

MR imaging could therefore improve diagnostic accuracy after therapy. It could play an important role in disease monitoring, especially in patients who are difficult to examine (i.e., children and dentophobic patients). Considering the high portion of intraosseous MR changes at non-healed sites, osseous edema could also guide clinicians to examine affected teeth further [11]. An important side effect of visualization of the disease is improving the patients’ motivation for reasonable compliance by seeing the direct results of the treatment.

To improve the diagnostic value of MRI, further information on a correlation between specific histopathological changes and intraosseous edema within the tooth-supporting bone is mandatory. Osseous edema is correlated histologically with the replacement of bone marrow fat by inflammatory infiltration, for instance, in rheumatoid arthritis [12]. However, bony changes in periodontal disease need further clarification. It will be interesting to see in further studies whether bone edema will continue to decrease in height and volume over a more extended period of time, as it is known that healing as reflected by the reduction of probing pocket depth occurs over a time period of 9 months following treatment of periodontal pockets [13]. Also, it will be interesting to see how skeletal renewal could be affected by bisphosphonates, denosumab, or antiangiogenetic drugs, which were exclusion criteria in this study.

There are limitations to our study that must be considered for the interpretation of the results:

- First, this study was designed as a prospective cohort study. With this study design, we cannot rule out alterations of periodontal edema occurring within the course of the disease without therapy. Therefore, we put our results in context with the diagnostic gold standard, the standardized clinical exam.
- Second, common restorative materials and resulting susceptibility artifacts can significantly reduce image quality in dental MRI. [3] With our sequences, we could use the majority of teeth for diagnostic evaluation (Table 1).
- Third, MRI soft tissue and intraosseous inflammatory changes are not specific for periodontitis. STIR hyperintense edema can also be caused, for instance, by primary inflammatory conditions, excessive functional (occlusal) stress, chronic inflammation, or previous endodontic or surgical treatment. [14, 15] For our study, we excluded patients who underwent surgery < 12 months before the first scan. However, at some sites, occlusal forces associated with primary or secondary malposition of teeth might have been at least partially responsible for the manifestation of osseous edema.
- Fourth, Gingival inflammation can be challenging to distinguish from bone edema through MR imaging by solely using the STIR sequences. The combination with the 3D T1 Black bone sequence is recommended for diagnostic accuracy.
- Fifth, the volumetric analysis through subtraction images is a limited method to compare bone marrow edema volumes. We observed an overall reduction of edema volume in jaw quadrants, in which bone marrow edema was present at the timepoint of the initial diagnosis. For more data confirming our results further studies using semiautomatic segmentation methods are needed.

In patients with periodontitis, MR T2 STIR can be used to monitor the bone marrow after periodontal treatment. MRI imaging provides thereby a novel tool to monitor the post-treatment course of the disease.

### Supplementary Information

Below is the link to the electronic supplementary material.

Supplementary file1 (PDF 52 KB)

## Abbreviations

BOP: Bleeding on probing
ED: Bone Marrow Edema depth
IQR: Interquartile range
PPD: Probing pocket depth
SEM: Standard error of mean
STIR: Short tau inversion recovery

## Declarations

### Guarantor

The scientific guarantor of this publication is Monika Probst.

### Statistics and biometry

No complex statistical methods were necessary for this paper.

### Study subjects or cohorts overlap

Some study subjects or cohorts have been previously reported in Probst M, Burian E, Robl T et al (2021) Magnetic resonance imaging as a diagnostic tool for periodontal disease: A prospective study with correlation to standard clinical findings—Is there added value? *J Clin Periodontol* 48:929-948

### Methodology

• prospective

• diagnostic or prognostic study

• performed at one institution
