# Supplementary material for: MRI-detected intraosseous bone marrow edema recedes after effective therapy of periodontitis
Source: Eur Radiol. 2023 Oct 19;34(5):3115–22. doi: 10.1007/s00330-023-10327-6 (PMC11126442; doi:10.1007/s00330-023-10327-6)
Supplement: Supplementary file 1 — Supplementary file1 (PDF 52 KB) [file 330_2023_10327_MOESM1_ESM.pdf]

Table S1 – Imaging parameters

Parameter	3D-T2-STIR	3D-FFE-T1-Black-Bone
Field of View	200 mm	180 mm
Matrix	308 x 308	420 x 419
Acquisition Voxel	0,65 x 0,65 x 1 mm^3	0,43 x 0,43 x 0,5 mm^3
Number of averages	1	1
Repetition time (TR)	2300 ms	10 ms
Echo time (TE)	184 ms	1,75 ms
Inversion Recovery (IR)	250 ms	
Gap	-0,5 mm	-0,25 mm
Slice oversample Factor	1,5	
Compressed-SENSE	yes	yes
Reduction	5	2,3
Water fat shift, WFS (pix) /Bandwidth (Hz)	1766/246	1503/289
